# A Turn-On Fluorescent Probe for Highly Selective Detection and Visualization of Hydrogen Sulfide in Fungi

**DOI:** 10.3390/molecules29030577

**Published:** 2024-01-24

**Authors:** Qingsong Yan, Shengui He, Lei Feng, Ming Zhang, Chaoyan Han, Yuzhuo Wu, Chao Wang, Xiaochi Ma, Tonghui Ma

**Affiliations:** 1 School of Medicine, College of Pharmacy, Nanjing University of Chinese Medicine, Nanjing 210023, China; 2 State Key Laboratory of Fine Chemicals, Dalian University of Technology, Dalian 116024, China; heshengui@mail.dlut.edu.cn (S.H.); mingzhng@sina.com (M.Z.); 3 Second Affiliated Hospital, Dalian Medical University, Dalian 116023, China; leifeng@dmu.edu.cn (L.F.); wuyuzhuo54@163.com (Y.W.); maxc1978@613.com (X.M.); 4 College of Pharmacy, College of Integrative Medicine, Dalian Medical University, Dalian 116044, China; hanacyy@163.com

**Keywords:** hydrogen sulfide, fluorescent probe, fungi, fluorescence imaging

## Abstract

Hydrogen sulfide (H_2_S) is a significant actor in the virulence and pathogenicity of fungi. The analysis of endogenous H_2_S in fungi benefits the prevention and treatment of pathogenic infections. Herein, a H_2_S-activated turn-on fluorescent probe named **DDX-DNP** was developed for the sensitive and selective detection of H_2_S. Using **DDX-DNP**, the ability of several oral fungi strains to produce H_2_S was identified, which was also validated using a typical chromogenic medium. In addition, **DDX-DNP** was successfully used for the visual sensing of endogenous H_2_S in fungal cells via microscope, flow cytometry, and colony imaging, along with a specific validation with the co-incubation of H_2_S production inhibitors in living cells. Above all, **DDX-DNP** could be used for H_2_S detection, the fluorescent imaging of fungi, and even the identification of related fungi.

## 1. Introduction

Hydrogen sulfide (H_2_S) was the third important signaling gas molecule discovered in the body, after carbon monoxide (CO) and nitric oxide (NO), and it has an unpleasant smell of rotten eggs [1,2]. Generally, H_2_S can be produced by enzymatic or non-enzymatic reactions. Now, several main enzymes related to the production of H_2_S have been characterized in mammals and bacteria, such as cystathionine-*γ*-lyase (CSE), cystathionine-*β*-synthase (CBS), cysteine aminotransferase (CAT), and 3-mercaptopyruvate sulfurtransferase (3-MST) [3,4,5,6]. CSE and CBS produce H_2_S through the condensation of homocysteine and cysteine or the *α*,*β*-elimination of cysteine, while 3-MST reductively converts 3-mercaptopyruvate to H_2_S [2,4,5]. In particular, in *Candida albicans*, CYS4 is annotated as encoding CBS for the synthesis of H_2_S in the *Candida* Genome Database [4]. On the other hand, polysulfides and glutathione could be converted to H_2_S via the non-enzymatic pathway [6]. As a well-known endogenous gas molecule, H_2_S has been known to exert all kinds of biological functions, such as relaxing vascular smooth muscles, mediating neuronal transmission, and regulating inflammation [7,8,9]. However, an abnormal level of H_2_S in biosystems could induce an array of malignant diseases including Down Syndrome, Alzheimer’s disease, and diabetes, as well as accelerate the proliferation and migration of cancer cells [10,11,12,13,14]. In addition, H_2_S, a volatile sulfur compound, is also one of the main causes of oral malodor [15]. Excessive H_2_S may also irritate the eyes and respiratory tract, resulting in the severe loss of consciousness, respiratory failure, and even death [16].

Given the biological importance of H_2_S, in the past few years, several methods have been reported for H_2_S detection and analysis, such as colorimetry, electrochemical assay, and gas chromatography [17,18,19]. Unfortunately, few of these techniques are suitable for living systems or for the in situ monitoring and analysis of H_2_S. On the contrary, compared with the traditional methods, small molecular fluorescent probes have attracted considerable attention because of their inherent advantages, for example, excellent selectivity, high sensitivity, real-time monitoring, nondestructive visualization, convenience, and so on [20,21,22,23,24,25].

Some fluorescent probes have been developed for the detection of H_2_S based on a variety of strategies, such as (1) azides and nitro compounds being reduced to amines, (2) the Michael addition reaction of the H_2_S-specific cleavage of the disulfide bond, nucleophilic reaction, and thiolysis reaction, and (3) the H_2_S-induced metal displacement approach and the metal indicator displacement approach [26,27,28,29,30,31]. These probes can image H_2_S in living cells, tissue, and blood samples in vivo, even in subcellular targeting location analysis [32,33,34,35]. Although great advancements have been made, unfortunately, none of them has been used in microorganisms, including for fungi detection and analysis. Hence, the design and synthesis of a highly selective fluorescent probe for monitoring H_2_S in fungi is of great importance and urgently desirable to better understand the detailed physiological and pathological effects of H_2_S.

In view of this, we designed and synthesized a fluorescent probe named **DDX-DNP** for the detection of H_2_S in fungi based on the thiolysis of the dinitrophenyl ether strategy [31,36,37]. Further research shows that the probe **DDX-DNP** has excellent optical performance, including biological applicability and good selectivity. More importantly, this probe successfully performed the visual sensing of endogenous H_2_S in fungi. To the best of our knowledge, this technique is a pioneering and effective tool for assessing H_2_S changes in the field of pathogenic infections.

## 2. Results and Discussion

### 2.1. Design of **DDX-DNP** and H_2_S Response

**Scheme 1:**
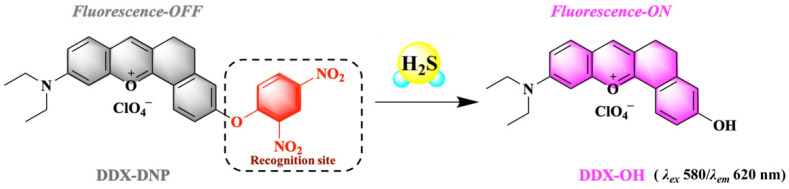
Schematic illustration of **DDX-DNP** detection of H_2_S.

To achieve the detection and imaging of H_2_S, in consideration of its dual nucleophilicity toward H_2_S, we introduced the 2,4-dinitrophenyl (DNP) group, which not only acts as a recognition site but also an effective fluorescence quencher [36]. The final structure of the designed fluorescent probe is shown in Scheme 1. In the absence of H_2_S, the probe **DDX-DNP** exhibited a weak fluorescence signal. When **DDX-DNP** reacted with H_2_S, the DNP group was removed from **DDX-DNP** so as to release the **DDX-OH**, exhibiting a remarkable fluorescence signal at 620 nm. Thus, H_2_S was detected qualitatively by recording the fluorescence intensity changes from probe **DDX-DNP** to the fluorophore, **DDX-OH**.

### 2.2. Spectral Characteristics and DFT Calculations of **DDX-DNP** and **DDX-OH**

**Figure 1:**
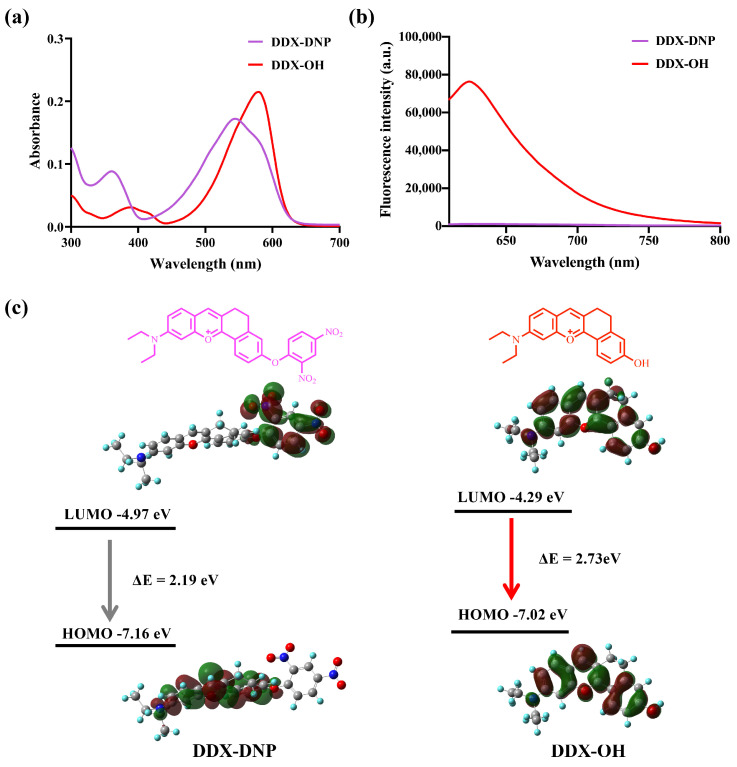
The absorption (**a**) and fluorescence (**b**) spectra of **DDX-DNP** (10 μM) and **DDX-OH** (10 μM) in KH_2_PO_4_–K_2_HPO_4_ buffer (50 mM, pH 7.4), gain = 80 (**b**); (**c**) density functional theory (DFT)–based optimized structures and frontier molecular orbitals (MOs) of **DDX-DNP** and **DDX-OH**.

In order to evaluate the detection properties of **DDX-DNP**, first, the optical spectral characteristics of **DDX-DNP** and the product, **DDX-OH**, were recorded using UV-vis absorbance and fluorescence spectroscopy. As shown in Figure 1a, **DDX-DNP** and **DDX-OH** displayed the maximum absorbance at 540 nm and 580 nm, respectively. Excited by laser at 580 nm, **DDX-OH** showed a strong fluorescence intensity at 620 nm, while **DDX-DNP** showed no fluorescence signal (Figure 1b). In addition, we also recorded the absorbance spectra of **DDX-DNP** at different concentrations (0–10 μM), and a good linear relationship was observed between the **DDX-DNP** concentrations and the absorbance values (Figure S1).

In order to gain a better insight into the fluorescence response behavior of **DDX-DNP** from the perspective of the molecular orbital, the geometries of **DDX-DNP** and **DDX-OH** were optimized, and their frontier relevant molecular energy levels were calculated as shown in Figure 1c [36,38]. The π electrons on the HOMO level of fluorescent probe **DDX-DNP** were mainly concentrated on the dihydrobenzo[c]xanthen skeleton. In contrast, on the LUMO level, these electrons were mainly distributed in the DNP group. This phenomenon means that a possible photoinduced electron transfer (PET) process occurs in **DDX-DNP**; the electron transfer from the dihydroben-zo[c]xanthen skeleton to the DNP group leads to a fluorescence “turn off” state. Nevertheless, the π electrons of the **DDX-OH** fluorophore were mainly delocalized in the whole-molecule skeleton on both the LUMO and HOMO levels, which resulted in a typical intramolecular charge transfer (ICT) process, and the strong fluorescence signal was “turned on”. In addition, the calculated energy gaps (LUMO → HOMO) of **DDX-DNP** and **DDX-OH** were 2.19 eV and 2.73 eV, respectively.

**Figure 2:**
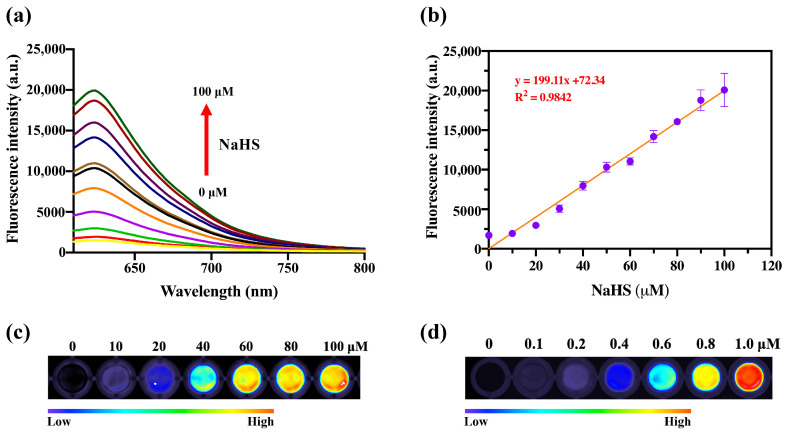
(**a**) Fluorescence spectra of reaction solutions of **DDX-DNP** (10 μM) with NaHS at different concentrations (0–100 μM) and (**b**) the linear relationship between fluorescence intensity at 620 nm and NaHS concentrations. (**c**) Fluorescence imaging of NaHS at different concentrations (0–100 μM) using **DDX-DNP** (10 μM) and (**d**) **DDX-OH** (0–1 μM) in 96 plates. The time for the incubation was 1 h, at 37 °C in KH_2_PO_4_-K_2_HPO_4_ buffer (50 mM, pH 7.4) (**a**,**c**). *λ*_ex_/*λ*_em_ = 580/620 nm.

Next, the fluorescence response of **DDX-DNP** toward H_2_S at different concentrations (0–100 μM) was also investigated. As can be seen in Figure 2a, along with the addition of incremental amounts of H_2_S, the fluorescence intensity enhancement at 620 nm, in a concentration-dependent manner, was observed clearly, which indicated that the DNP moiety was cut off by H_2_S to induce a dramatic and rapid fluorescence turn-on response. A point worth emphasizing is that a good linear relationship (R^2^ = 0.9937) was observed between the H_2_S concentrations and the fluorescence intensity at 620 nm (Figure 2b). Furthermore, we measured the visual fluorescence images with a series of concentrations for H_2_S (0–100 μM) and **DDX-OH** (0–1 μM) in 96 plates, which showed that the fluorescence intensity gradually increased with the increasing H_2_S concentration (Figure 2c,d), indicating the potential visual detection of H_2_S. Above all, **DDX-DNP** could be activated by H_2_S, suggesting its application as a turn-on fluorescent probe for H_2_S.

**Figure 3:**
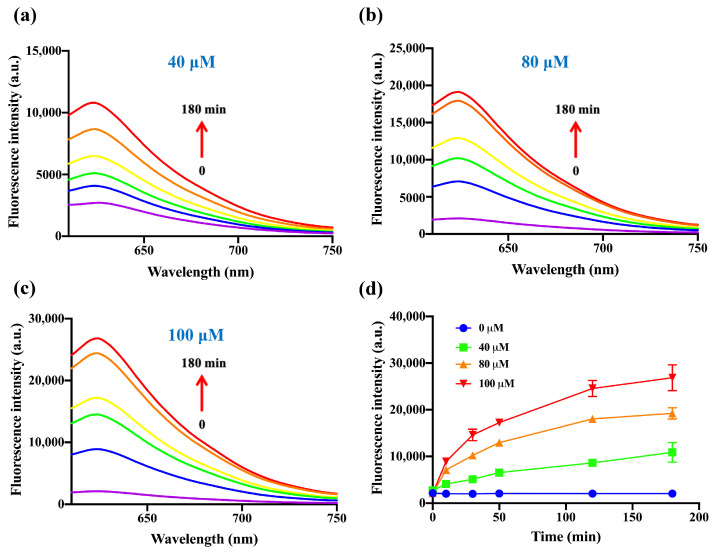
The fluorescence spectra of **DDX-DNP** (10 μM) toward NaHS 40 μM (**a**), 80 μM (**b**), and 100 μM (**c**) with different incubation times (0–180 min). (**d**) Time-dependent fluorescence intensity of **DDX-DNP** in the presence of various concentrations of NaHS (0, 40, 80, and 100 μM, respectively). *λ*_ex_/*λ*_em_ = 580/620 nm.

On the other hand, the fluorescence response of **DDX-DNP** toward H_2_S with different incubation times, in the range of 0–180 min, was also explored. With the increase in incubation time, the fluorescence intensity at 620 nm showed an increasing tendency, and it appeared to be flat after 120 min (Figure 3). Therefore, we finally identified 100 μM of H_2_S and an incubation time of 60 min as the key factors for the H_2_S fluorescence detection system based on the above results. In addition, kinetic analysis (Figure S3) revealed that the apparent rate constant *k′* for the reaction of **DDX-DNP** with H_2_S was 0.023 min^−1^, and the pseudo-first-order rate constant *k* for the reaction of **DDX-DNP** with H_2_S was 0.23 M^−1^ min^−1^.

Job’s Plot [39] was generated by continuously varying the mole fraction of NaHS from 0 to 1 in a solution of NaHS + **DDX-DNP** with a total concentration of 100 μM. The fluorescence intensity became the strongest when the molar fraction of NaHS was 0.5, which indicated that the binding stoichiometry between probe **DDX-DNP** and NaHS is 1:1 (Figure S4).

### 2.3. Selectivity of **DDX-DNP** for the Detection of H_2_S

**Figure 4:**
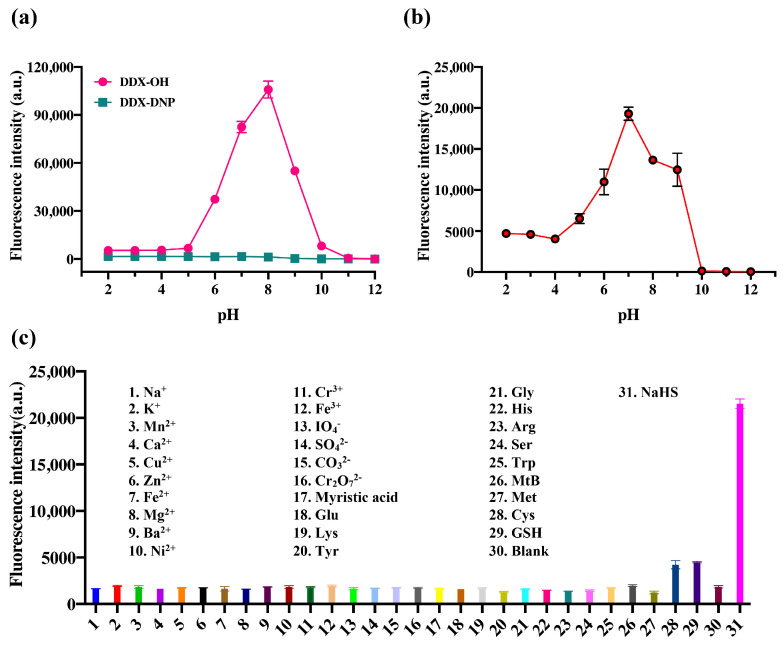
(**a**) Fluorescence intensity of **DDX-DNP** (10 μM) and product **DDX-OH** (10 μM) in various phosphate solutions at different pHs (2–12), gain = 85%. (**b**) The reaction rate of **DDX-DNP** (10 μM) toward H_2_S in various phosphate buffers with different pH values (2–12). (**c**) Fluorescence signal changes in **DDX-DNP** (10 μM) in phosphate buffer upon the sequential addition of various species. The time for the incubation was 1 h (**b**,**c**). All species’ concentrations were 100 μM. *λ*_ex_/*λ*_em_ = 580/620 nm.

In this study, the fluorescence intensities of **DDX-DNP** and **DDX-OH** under different pH conditions were determined and analyzed. As shown in Figure 4a, **DDX-DNP** showed almost no variation from pH 2.0 to 12.0, and **DDX-OH** with the hydroxyl group exposed to the outside manifested a strong fluorescence signal from pH 6.0 to 9.0.

In order to compare the reaction rates at different pH values and confirm whether the probe could function smoothly under the studied physiological conditions, the fluorescence intensity of probe **DDX-DNP** toward H_2_S was investigated at different pH values (Figure 4b). The probe **DDX-DNP**’s fluorescence intensity was enhanced noticeably from pH 4 to 9, and it reached the maximum fluorescent intensity at pH 7, while the other pH values did not exhibit any obvious fluorescence enhancement. In fact, fluorescence quenching occurred under strong alkali conditions. Therefore, pH 7.4 was determined to be the optimal pH for the incubation system. The incubation temperature was also measured, as shown in Figure S5. The reaction rate reached its maximum at 37 °C, and it did not continue to accelerate with the increasing temperature.

The excellent selectivity of the fluorescent probe for the target substance is an essential condition for its practical application. Thereby, we further explored the selectivity of **DDX-DNP** toward H_2_S. As shown in Figure 4c, in the presence of various substances such as common metal ions, Na^+^, K^+^, Mn^2+^, Ca^2+^, Cu^2+^, Zn^2+^, Fe^2+^, Mg^2+^, Ba^2+^, Ni^2+^, Cr^3+^, Fe^3+^, IO_4_^−^, SO_4_^2−^, CO_3_^2−^, and Cr_2_O_7_^2−^, and natural amino acids, Glu, Lys, Tyr, Gly, His, Arg, Ser, Trp, and biothiols (Cys, Met, GSH), the fluorescence intensity of **DDX-DNP** was measured. Notably, only glutathione and cysteine could trigger a weak fluorescence signal enhancement and cause hardly any interference in H_2_S detection. Other species could not render an apparent change in fluorescence intensity, which demonstrated that **DDX-DNP** displayed a good selectivity for the detection of endogenous H_2_S.

### 2.4. Confirmation about the Reaction between **DDX-DNP** and H_2_S

In order to confirm the sensing mechanism of **DDX-DNP** for H_2_S, HPLC-combined electrospray ionization–mass spectrometry (ESI-MS) analysis experiments were performed. As illustrated in Figure S11, after incubation with NaHS, a new ion peak at *m*/*z* 320.40 was observed, which could be assigned to the product **DDX-OH** [M − H]^−^. Therefore, the reaction mechanism of **DDX-DNP** for sensing H_2_S is proposed, as shown in Scheme S2.

Moreover, the chromatograms were also recorded using LC–MS/MS data for the fluorescent probe **DDX-DNP**, product **DDX-OH**, and reaction solution. As shown in Figure S6, a chromatographic peak of the probe **DDX-DNP** at the retention time of 3.44 min was observed, and **DDX-DNP** displayed a narrow peak at 3.29 min. Upon the addition of NaHS, a peak at 3.44 min (**DDX-DNP**) along with a peak at 3.29 min (**DDX-OH**) was recorded. Altogether, these results convey that the H_2_S-triggered thiolysis of dinitrophenyl ether occurred, releasing the target fluorophore.

### 2.5. High-Throughput Screening for Fungi-Produced H_2_S from Tongue-Coating Fungi Strains Based on **DDX-DNP**

**Figure 5:**
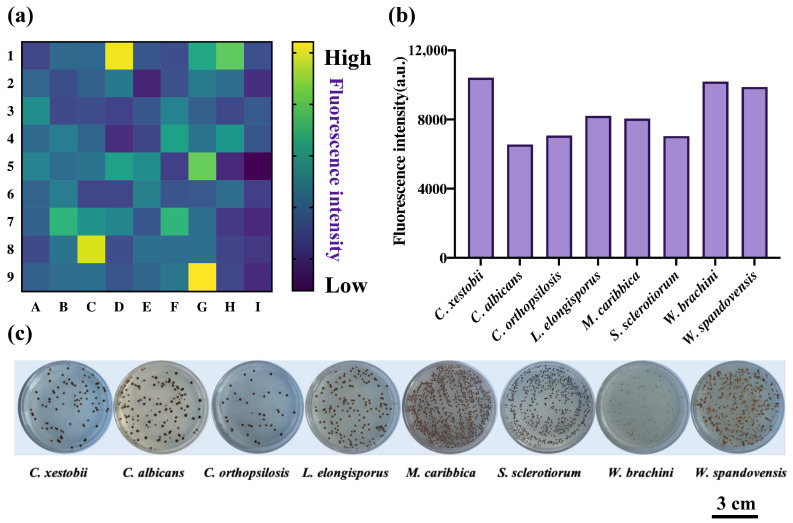
(**a**) The heat map of high-throughput screening of H_2_S-producing strains of tongue-coating fungi generated using **DDX-DNP** (25 μM); (**b**) the quantization results of 8 potential yeast strains with high yields of H_2_S; and (**c**) the visual detection of H_2_S generation of 8 yeast strains using BiGGY agar plates.

As it is well known, redox homeostasis is essential for host colonization by pathogens, while H_2_S has antioxidative properties and a protective bacterial effect against antibiotics [4], thus further aggravating the pathogenicity of pathogens. H_2_S is not only generated by bacteria, but also it has been previously reported that some fungi can produce H_2_S. In our previous work, we leveraged the cultivation and sequencing of tongue-coating samples from healthy individuals to create a massive fungi bank. Thus, we created sufficient and convenient fungi strains for the high-throughput screening of fungi-produced H_2_S based on **DDX-DNP**. As displayed in Figure 5a,b, eight yeast strains were identified with high yields of H_2_S production from tongue-coating fungi, among which the *Candida xestobii* and *Wickerhamiella spandovensis* strains were the most active H_2_S-producing strains. *Candida albicans*, as a pathogen, was also previously discovered to be a H_2_S-producing fungus [4].

In addition, we used the BiGGY (bismuth sulfite glucose glycine yeast) agar plate to verify the production of H_2_S by these identified yeasts. Before the formal experiments, first, we measured the selectivity of the BiGGY medium toward H_2_S. In the medium, bismuth ammonium citrate could be reduced by the H_2_S donor NaHS to yield a brownish-black color, while failing to react with glutathione, methionine, cysteine, and other thiol-containing compounds (Figure S7). Therefore, this assay acts as a specific indicator to evaluate the H_2_S production of fungi. As we expected, when the identified eight yeast stains were grown in the BiGGY agar, the brownish-black color of the colonies appeared (Figure 5c), which was consistent with the results identified using **DDX-DNP**, further indicating that the developed fluorescent probe was feasible for detecting H_2_S.

### 2.6. Fluorescence Imaging of Fungi via H_2_S Monitoring

**Figure 6:**
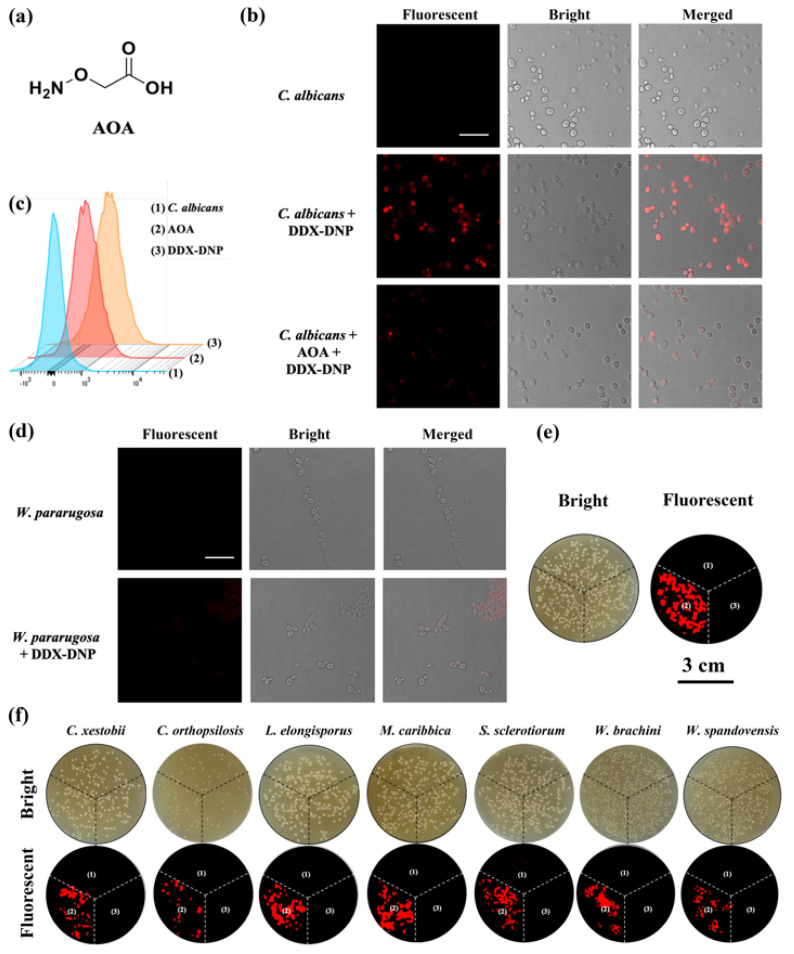
(**a**) Chemical structure of the CBS inhibitor AOA; (**b**) fluorescence images of *C. albicans* incubated with **DDX-DNP** (25 μM) and in the presence of inhibitor AOA, scale bar = 25 μm; (**c**) flow cytometric analysis *of C. albicans* (blue) with **DDX-DNP** (orange) or both AOA and **DDX-DNP** (red); (**d**) fluorescence images of *W. paraugosa* incubated with **DDX-DNP**, scale bar = 20 μm; and (**e**,**f**) fluorescence images of yeast colonies on MtB agar plates along with bright fields. The plate was divided into three sections: (1) yeast colonies as the blank section in absence of **DDX-DNP**; (2) yeast colonies incubated with 25 μM **DDX-DNP**; and (3) yeast colonies incubated with 1 mM AOA and 25 μM **DDX-DNP**.

Inspired by its satisfying sensing performance and good selective ability, the sensing of endogenous H_2_S in fungi by **DDX-DNP** was performed with validation using 2-(Aminooxy) acetic acid (AOA) as the inhibitor of H_2_S production in cells. Then, a fungi imaging experiment was carried out using *Candida albicans*. As shown in Figure 6b, a non-fluorescence signal was observed in the blank group, and a strong fluorescence signal was detected for fungal cells after the co-incubation of **DDX-DNP**, which indicated the abundant generation of endogenous H_2_S in *Candida albicans.* Furthermore, in the presence of AOA, the *Candida albicans* cells co-incubated with **DDX-DNP**, showing weak fluorescence intensity, which validated the specific detection of endogenous H_2_S by **DDX-DNP**. Subsequently, the ability of the fluorescent probe **DDX-DNP** to detect endogenous H_2_S in *Candida albicans* was also verified using a flow cytometric assay. As shown in Figure 6c, compared with the blank group (blue line), *Candida albicans* incubated with **DDX-DNP** displayed a significant shift (orange line); however, the shift in the inhibitor group (red line) was clearly reduced. At the same time, we also selected a fungus, *W. paraugosa*, which released almost no H_2_S as a negative strain for fluorescence imaging experiments. There is no doubt that no fluorescence intensity was observed in its fluorescence field (Figure 6d), which was in accordance with the results obtained in the fungi solution detection (Figure 5b). This further demonstrates the highly selective characteristic of endogenous H_2_S sensing using **DDX-DNP**. Additionally, the fluorescence imaging of *Candida albicans* and other yeast strains was also determined using agar plates. The fungal colonies displayed a significant fluorescence signal after being incubated with **DDX-DNP**, and the signal was measured using the Typhoon imager (Figure 6e,f). Thus, **DDX-DNP** has good biocompatibility and can achieve the detection and visualization of endogenous H_2_S created by living fungi, which suggests its potential applications for fungi identification or activity assays regarding H_2_S production.

## 3. Materials and Methods

### 3.1. Materials and Instruments

Unless otherwise noted, all the chemical reagents and solvents were purchased from commercial suppliers Maclin Biochemical Co., Ltd. (Shanghai, China) and Kermel Chemical Reagent Co., Ltd. (Tianjin, China) and used without further refinement. Pepton, yeast extract, and agar were purchased from Solarbio Science & Technology Co., Ltd. (Beijing, China). BiGGY (bismuth sulfite glucose glycine yeast) agar medium was obtained from Hope Bio-Technology Co., Ltd. (Qingdao, China).

^1^H-NMR and ^13^C-NMR spectra of synthesized products were measured on a Bruker-600 spectrometer (Bruker, Madison, WI, USA), using tetramethylsilane (TMS) as an internal standard. High-resolution mass spectra (HRMS) of synthesized products were measured using an AB SciexX500r TOF mass spectrometer (AB SCIEX, Framingham, MA, USA). The fluorescence microscopic images of fungal cells were obtained using a confocal microscope (Leica, Wetzlar, Germany). The fluorescence images of fungous colonies on agar plates were recorded using a fluorescence image analyzer (Amersham Typhoon, Tokyo, Japan). The bioassay solutions in 96-well plates were also analyzed using an microplate reader (Tecan, Männedorf, Switzerland). All pH measurements were taken using a pHS-3C pH meter (INESA, Shanghai, China).

### 3.2. Synthetic Procedure of Probe **DDX-DNP**

The fluorescent probe **DDX-DNP** was synthesized according to the methodology shown in Scheme S1, and the NMR and HRMS data are provided in the Supplementary Materials.

#### 3.2.1. Synthesis of **DDX-OH**

**DDX-OH** was synthesized according to a previously reported procedure [40]. 4-(Diethylamino)salicylaldehyde (1.93 g, 10 mmol) and 6-Hydroxy-1-tetralone (1.62 g, 10 mmol) were dissolved in concentrated sulfuric acid (10 mL), and the solution was stirred at 90 °C in an argon atmosphere for 6 h. The solution was cooled to room temperature and then poured into ice water (100 mL) containing HClO_4_ (4 mL). The mixture was filtered to obtain the product **DDX-OH** as a red solid (2.15 g, 51.2%), which was used for the next step without further purification. ^1^H NMR (500 MHz, DMSO-*d*_6_) *δ*_H_ 8.62 (s, 1H), 8.16 (d, *J* = 8.7 Hz, 1H), 7.91 (d, *J* = 9.4 Hz, 1H), 7.41 (dd, *J* = 9.3, 2.3 Hz, 1H), 7.27 (d, *J* = 2.0 Hz, 1H), 6.94 (dd, *J* = 8.7, 2.3 Hz, 1H), 6.87 (d, *J* = 2.2 Hz, 1H), 3.67 (q, *J* = 7.0 Hz, 4H), 3.01 (s, 4H), 1.24 (t, *J* = 7.1 Hz, 6H).

#### 3.2.2. Synthesis of **DDX-DNP**

**DDX-OH** (210 mg, 0.50 mmol), 2,4-dinitrofluorobenzene (93 mg, 0.50 mmol), and K_2_CO_3_ (207 mg, 1.5 mmol) were dissolved in CH_3_CN (20 mL), and the solution was stirred at room temperature in an argon atmosphere for 1 h. The mixture was evaporated under reduced pressure, and the crude product was purified using silica gel chromatography (CH_2_Cl_2_: MeOH = 15:1, *v:v*) to obtain the product **DDX-DNP** as a red solid (150 mg, 51.1%). ^1^H NMR (600 MHz, CDCl_3_) *δ*_H_ 8.88 (d, *J* = 2.6 Hz, 1H), 8.51 (s, 1H), 8.48 (dd, *J* = 9.4, 2.6 Hz, 1H), 8.40 (d, *J* = 8.6 Hz, 1H), 7.90 (d, *J* = 9.4 Hz, 1H), 7.38 (d, *J* = 9.1 Hz, 1H), 7.25 (dd, *J* = 9.6, 2.2 Hz, 1H), 7.17 (dd, *J* = 8.7, 2.2 Hz, 1H), 7.12 (d, *J* = 2.2 Hz, 1H), 7.07 (d, *J* = 2.2 Hz, 1H), 3.69 (m, 4H), 3.13 (m, 4H), 1.37 (t, *J* = 7.0 Hz, 6H). ^13^C NMR (150 MHz, CDCl_3_) *δ*_C_ 162.0, 159.2, 159.0, 156.2, 153.7, 148.9, 144.9, 143.0, 140.6, 132.6, 129.6, 129.3, 123.7, 122.1, 121.8, 121.1, 119.6, 119.3, 118.8, 118.3, 96.2, 46.5, 27.1, 24.9, 12.7. HR-MS *m*/*z* 486.1645 (C_27_H_24_N_3_O_6_^+^), calcd for 486.1660.

### 3.3. Density Functional Theory (DFT) Calculations

All computations were performed in GaussView 6.0.16 using Gaussian 16. The B3LYP/6-31G(d) basis set method was applied to optimize the low-energetic conformations of **DDX-DNP** and **DDX-OH** using density functional theory (DFT). Then, the lowest unoccupied molecular orbital (LUMO) and highest occupied molecular orbital (HOMO) calculations were performed using Mos tool in GaussView 6.0.16, through a fchk-type file. The calculation was not limited by bonds/angles/dihedral angles, and all atoms could be freely optimized.

### 3.4. High-Performance Liquid Chromatography–Mass Spectrometry of **DDX-DNP** and **DDX-OH**

An HPLC–MS/MS-system Triple Quad 4500 MD (AB SCIEX, USA) was utilized to identify **DDX-DNP** and **DDX-OH** in the reacted solution. The MS parameters were optimized as follows: for **DDX-DNP**, Q1 Mass 487.1Da, Q3 Mass 440.0 Da, Declustering Potential (DP) 110.0 volts, collision energy (CE) 50.0 volts; and for **DDX-OH**, Q1 Mass 321.2 Da, Q3 Mass 277.0 Da, DP 110.0 volts, CE 53.0 volts. The mobile phase was composed of A (0.1% *v*/*v* formic acid aqueous solution) and B (acetonitrile). Next, the HPLC elution conditions were optimized as follows: 0–1 min: 90% A; 1–2.5 min: 90–0% A; 2.5–4 min: 0% A; 4.0–5.5 min: 0–90% A; and 5.5–6.5 min: 90% A. The flow rate and the column temperature were set to 0.3 mL/min and 40 °C, respectively. Subsequently, the solutions of **DDX-DNP** and **DDX-OH** and the reacted solution of **DDX-DNP** and NaHS were injected, respectively. Finally, all the data were collected and processed using Analyst 1.6.3 software.

### 3.5. Spectroscopic Measurements

Unless otherwise noted, all measurements were performed in KH_2_PO_4_-K_2_HPO_4_ buffer (50 mM, pH 7.4). H_2_S was prepared from NaHS, which is a common donor and a standard source for H_2_S. The stock solution of the probe **DDX-DNP** (25 mM) was prepared in DMSO and then diluted to the desired concentration prior to the next experiment. The fluorescence spectra of the resultant solution were recorded using the maximum absorbance wavelength of the corresponding fluorophores as the excited wavelength (**DDX-OH**: *λ*_ex_ = 580 nm).

Fluorescence response tests of **DDX-DNP** (10 μM) towards NaHS at different concentrations (0–100 μM) were performed in KH_2_PO_4_-K_2_HPO_4_ buffer for 60 min, incubated at 37 °C. Meanwhile, the fluorescence responses of **DDX-DNP** (10 μM) towards NaHS (0, 40, 80, and 100 μM) with different incubation times (0–180 min) were investigated in phosphate buffer, when incubated at 37 °C.

For the effect of pH on the fluorescence probe, different pH values of the phosphate buffer from 2.0 to 12.0 were adjusted by supplementing appropriate volumes of standard phosphoric acid or potassium hydroxide, which were used for the fluorescence intensity detection of **DDX-DNP**.

### 3.6. Kinetic Studies [38]

The reaction of **DDX-DNP** (10 μM) with H_2_S in KH_2_PO_4_-K_2_HPO_4_ buffer (50 mM, pH 7.4) at 37 °C was monitored by measuring the fluorescence intensity (*E*). The apparent rate constant for the reaction was determined by fitting the fluorescence intensity of the samples to the pseudo- first-order equation:

ln((*E*_max_ − *E_t_*)/*E*_max_) = −*k*′*t* (1)

where *E_t_* and *E*_max_ are the fluorescent intensities at 620 nm at times *t* and the maximum value obtained during the reaction. *k′* is the apparent rate constant. The pseudo-first-order rate constant *k* (M^−1^ s^−1^) was obtained from Equation (2),

*k*′ = *kC* (2)

where *C* is the concentration of H_2_S.

### 3.7. Selectivity Evaluation

The selectivity of **DDX-DNP** toward H_2_S and other species regarding the fluorescence intensity of **DDX-DNP** was evaluated in the presence of various ions and amino acids, such as NaCl, KCl, MnCl_2_, CaCl_2_, CuSO_4_, MgCl_2_, BaCl_2_, ZnCl_2_, FeCl_2_, FeCl_3_, CaCO_3_, KIO_4_, K_2_Cr_2_O_7_, tyrosine (Tyr), glycine (Gly), histidine (His), arginine (Arg), serine (Ser), tryptophan (Trp), glutathione (GSH), methionine (Met), lysine (Lys), cysteine (Cys), and myristic acid. All species’ concentrations were 100 μM.

### 3.8. Fungi Strains and Cultivation

All the yeast strains from a single colony were initially propagated in liquid martin broth modified medium (MtB), supplemented with or without 2% agar, at 32 °C in an orbital shaker (130 rpm) or a constant-temperature incubator. The yeast strains studied in this work are listed in Table S1. The fugal cells were cultured to an optical density at 600 nm (OD_600_) of 0.8 to 1.0, namely, culturing up to the logarithmic phase for experiments.

### 3.9. **DDX-DNP** High-Throughput Screening for H_2_S Production by Tongue-Coating Fungi

All the yeast strains were cultured in the MTB medium for at least 24 h to obtain enough fungal cells with an OD value of 1.0. Then, the probe **DDX-DNP** (25 μM) was added into the culture for co-incubation with yeast for about 4 h at 32 °C. After the fungal supernatants were harvested using centrifugation at 20,000× *g* for 10 min, the supernatant solutions were used to measure the fluorescence spectra using a microplate reader (*λ*_ex_ 580/*λ*_em_ 620 nm).

### 3.10. Fluorescence Imaging of Fungi via the Detection of H_2_S

The potential hydrogen sulfide-producing strains were cultured on MTB agar for 3–5 days. Fungal colonies were observed on the plates at 32 °C; then, the plates were divided into three regions, and **DDX-DNP** (25 μM) was sprayed on to the fungal colonies and incubated for 4 h at 32 °C. The inhibitor group were preincubated with AOA (1 mM) for 2 h at 32 °C. Finally, the plates were scanned using an Amersham Typhoon RGB (*λ*_ex_ = 532 nm).

*Candida albicans* and negative strains were cultured in the MTB medium; then, **DDX-DNP** was added into the medium with a final concentration of 25 μM and incubated at 32 °C for 4 h. The inhibitor group were preincubated with AOA (1 mM) for 2 h at 32 °C. After washing the whole cells three times with PBS, they were dropped into glass slides and immobilized with coverslips for imaging experiments. Finally, the imaging cells were observed using a Leica Confocal Microscope with *λ*_ex_ 561/*λ*_em_ 602–650 nm. The fluorescent signal was also recorded using flow cytometry. A total of about 10,000 events were collected for data analysis, without using specific gating policies. The data were analyzed using FlowJoversion v10.8.1 software.

## 4. Conclusions

To sum up, we designed and synthesized a turn-on fluorescent probe for monitoring H_2_S based on the H_2_S-triggered thiolysis of the dinitrophenyl ether strategy. In the presence of H_2_S, the fluorescent probe **DDX-DNP** exhibits a remarkable fluorescence enhancement at 620 nm. More importantly, **DDX-DNP** was the first fluorescent probe successfully used for the fluorescence bioimaging of H_2_S in fungi. In short, **DDX-DNP**, as a turn-on fluorescent probe activated by H_2_S, can be used for H_2_S detection, as well as for the visual sensing of endogenous H_2_S in living fungi.

## Data Availability

Data are contained within the article or Supplementary Material.

## Supplementary Materials

The following supporting information can be downloaded at: https://www.mdpi.com/article/10.3390/molecules29030577/s1.
