# Supplementary material for: A Turn-On Fluorescent Probe for Highly Selective Detection and Visualization of Hydrogen Sulfide in Fungi
Source: Molecules. 2024 Jan 24;29(3):577. doi: 10.3390/molecules29030577 (PMC10856155; doi:10.3390/molecules29030577)
Supplement: Supplementary file 1 [file molecules-29-00577-s001.zip › molecules-2787363-supplementary.pdf]

Supplementary Materials

A Turn-On Fluorescent Probe for Highly Selective Detection and Visualization of Hydrogen Sulfide in Fungi

Qingsong Yan [1,†], Shengui He [2,†], Lei Feng [3,4], Ming Zhang [2], Chaoyan Han [4], Yuzhuo Wu [3], Chao Wang [4,*], Xiaochi Ma [3] and Tonghui Ma [1,*]

[1] School of Medicine, College of Pharmacy, Nanjing University of Chinese Medicine, Nanjing 210023, China; yanqingsong1991@sina.com

[2] State Key Laboratory of Fine Chemicals, Dalian University of Technology, Dalian 116024, China; heshengui@mail.dlut.edu.cn (S.H.); mingzhng@sina.com (M.Z.)

[3] Second Affiliated Hospital, Dalian Medical University, Dalian 116023, China; leifeng@dmu.edu.cn (L.F.); wuyuzhuo54@163.com (Y.W.); maxc1978@613.com (X.M.)

[4] College of Pharmacy, College of Integrative Medicine, Dalian Medical University, Dalian 116044, China; hanacyy@163.com

* Correspondence: wach_edu@sina.com (C.W.); matonghui@njucm.edu.cn (T.M.)

† These authors contributed equally to this work.

Table of Contents

Scheme S1. The synthesis route of fluorescent probe **DDX-DNP**. S3

Scheme S2. The proposed sensing mechanism of **DDX-DNP** toward H_2S. S3

Figure S1. (a) The absorbance spectra of **DDX-DNP** at different concentrations (0.5, 1, 2, 4, 6, 8, 10 μM) in KH_2PO_4-K_2HPO_4 buffer and its linear relationship between the UV-absorbance and concentrations (b). S4

Figure S2. The fluorescence spectra of **DDX-DNP** (10 μM) with different incubation time (0-180 min) in KH_2PO_4-K_2HPO_4 buffer (50 mM, pH7.4) at 37 °C. λ_{ex}/ λ_{em} =580/620 nm. S4

Figure S3. Pseudo first-order kinetic plot of reaction of probe **DDX-DNP** (10 μM) with NaHS (100 μM) in KH_2PO_4-K_2HPO_4 buffer, for k = 0.23 M^{-1} min^{-1}. λ_{ex}/ λ_{em} =580/620 nm. S5

Figure S4. Job's plot of the **DDX-DNP**-NaHS complexes in the phosphate buffer, keeping the total concentration of **DDX-DNP** and NaHS at 100 μM. Emission wavelength was 580 nm. S5

Figure S5. Influence of incubation temperature on the reaction rate of H_2S (100 μM) with **DDX-DNP** (10 μM). The reaction was performed in KH_2PO_4-K_2HPO_4 buffer at different temperature for 60 min. λ_{ex}/λ_{em}=580/620 nm. S6

Figure S6. LC-MS chromatogram of **DDX-DNP**, **DDX-OH** and **DDX-DNP** reacted with NaHS for 60 min, mobile phase was acetonitrile/phosphate buffer =1/1 (*v/v*). S6

Figure S7. The BiGGY plate assay specifically for measurement of H_2S. Bismuth ammonium citrate in BiGGY medium can be reduced by H_2S donors NaHS and yield brown colors but fail to react with glutathione, L-homocysteine, L-methionine, L-cysteine and MtB medium. Exception for MtB medium (yeast extract 2g/L, peptone 5g/L, $MgSO_4$ 0.5g/L, KH_2PO_4 1g/L, glucose 20g/L), all species' concentrations were 100 μM. S7

Figure S8. ^{1}H NMR spectrum of **DDX-DNP**. S8

Figure S9. ^{13}C NMR spectrum of **DDX-DNP**. S8

Figure S10. HRMS of **DDX-DNP**. S9

Figure S11. ESI-MS spectra of **DDX-DNP** in the presence of excess amount of NaHS in the phosphate buffer (50 mM, pH 7.4) containing acetonitrile. S9

Table S1. The tongue coating fungi strains used in this study. S10

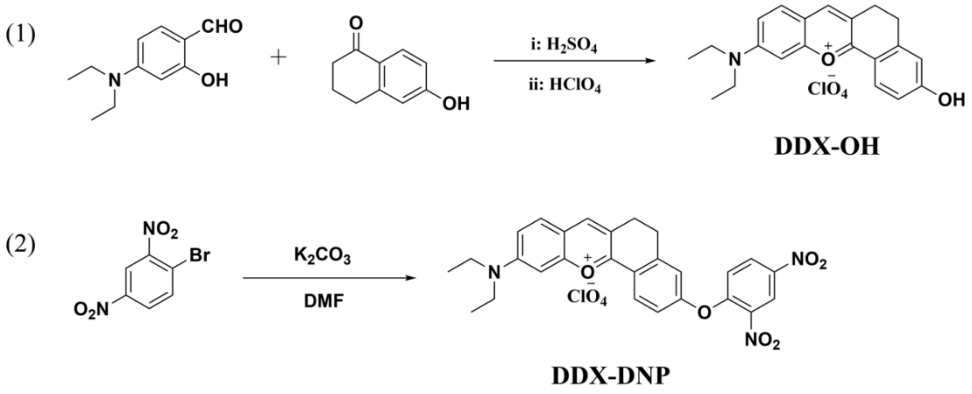

Scheme S1. The synthesis route of fluorescent probe **DDX-DNP**.

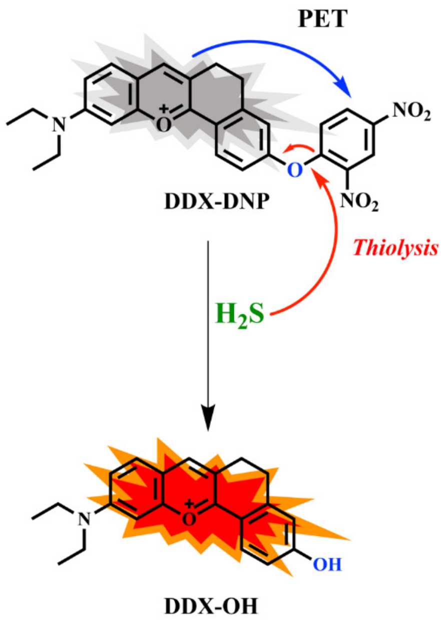

Scheme S2. The proposed sensing mechanism of **DDX-DNP** toward H_2S.

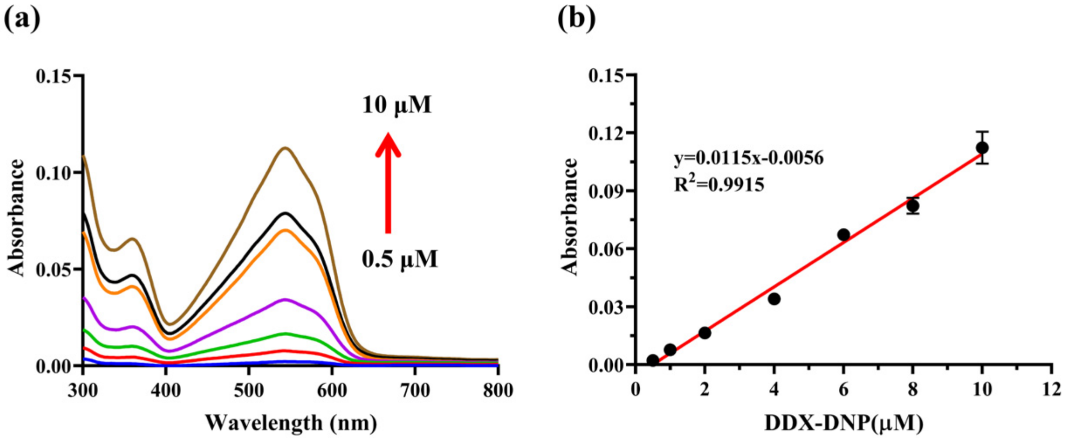

Figure S1. (a) The absorbance spectra of **DDX-DNP** at different concentrations (0.5, 1, 2, 4, 6, 8, 10 μM) in KH_2PO_4-K_2HPO_4 buffer and its linear relationship between the UV-absorbance and concentrations (b).

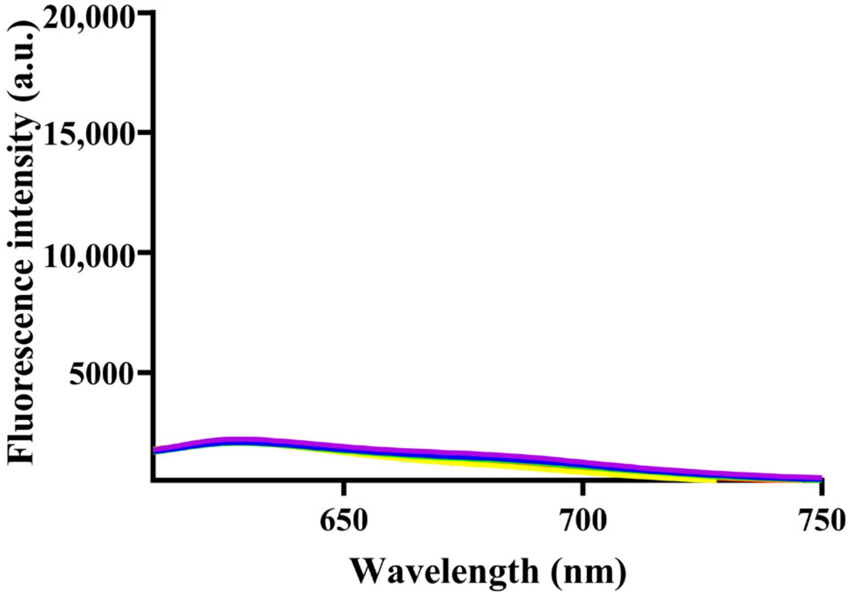

Figure S2. The fluorescence spectra of **DDX-DNP** (10 μM) with different incubation time (0-180 min) in KH_2PO_4-K_2HPO_4 buffer (50 mM, pH7.4) at 37 ℃. λ_{ex}/ λ_{em} =580/620 nm.

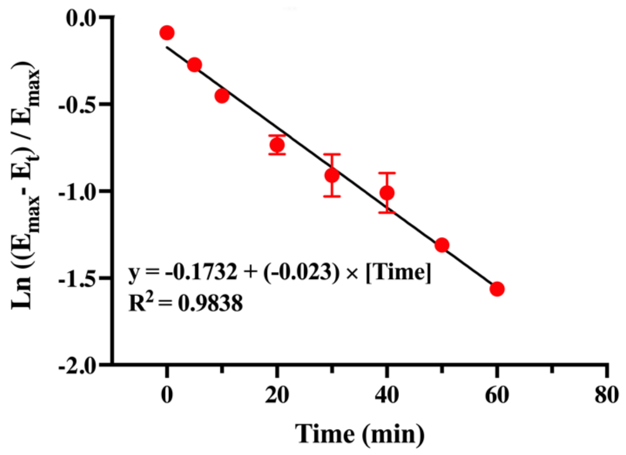

Figure S3. Pseudo first-order kinetic plot of reaction of probe **DDX-DNP** (10 μM) with NaHS (100 μM) in KH_2PO_4-K_2HPO_4 buffer, for k = 0.23 M^{-1} min^{-1}. λ_{ex}/ λ_{em} =580/620 nm.

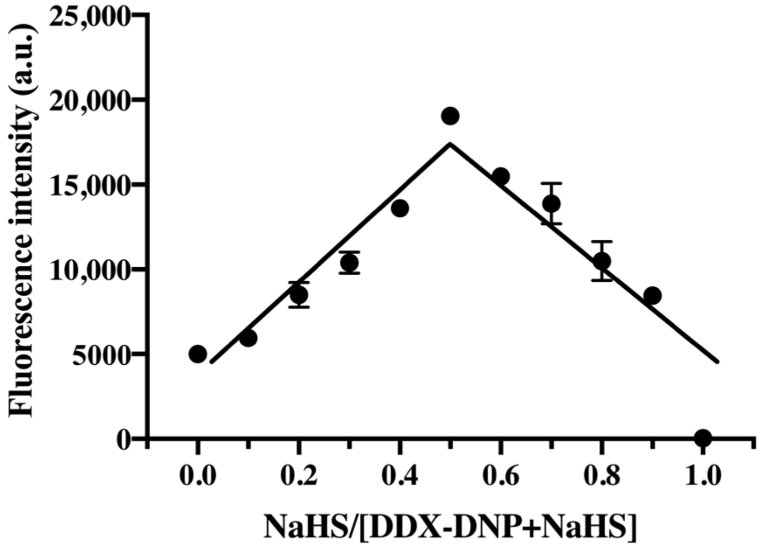

Figure S4. Job's plot of the **DDX-DNP**-NaHS complexes in the phosphate buffer, keeping the total concentration of **DDX-DNP** and NaHS at 100 μM. Emission wavelength was 620 nm.

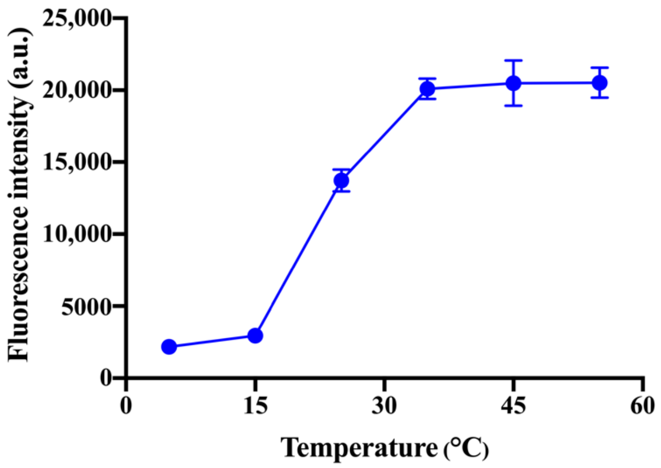

Figure S5. Influence of incubation temperature on the reaction rate of H_2S (100 µM) with **DDX-DNP** (10 µM). The reaction was performed in KH_2PO_4-K_2HPO_4 buffer at different temperature for 60 min. $\lambda_{ex}/\lambda_{em}$=580/620 nm.

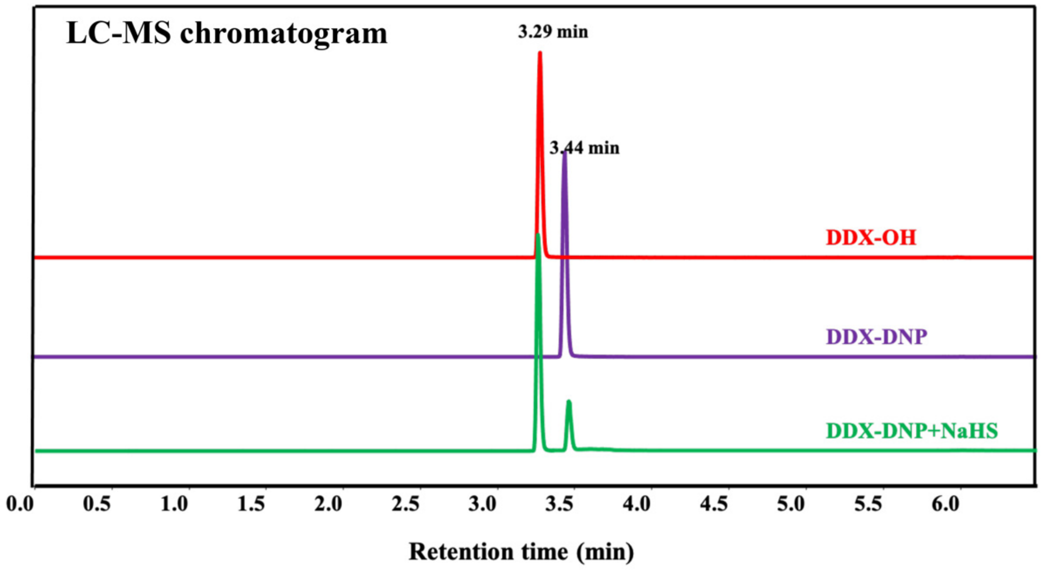

Figure S6. LC-MS chromatogram of **DDX-DNP**, **DDX-OH** and **DDX-DNP** reacted with NaHS for 60 min, mobile phase was acetonitrile/phosphate buffer =1/1 (*v/v*).

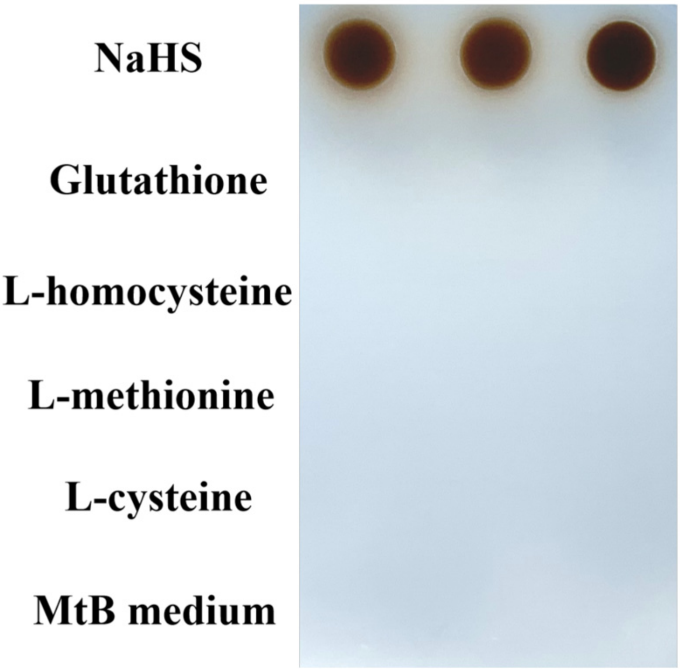

Figure S7. The BiGGY plate assay specifically for measurement of H_2S. Bismuth ammonium citrate in BiGGY medium can be reduced by H_2S donors NaHS and yield brown colors but fail to react with glutathione, L-homocysteine, L-methionine, L-cysteine and MtB medium. Exception for MtB medium (yeast extract 2g/L, peptone 5g/L, $MgSO_4$ 0.5g/L, KH_2PO_4 1g/L, glucose 20g/L), all species' concentrations were 100 μM.

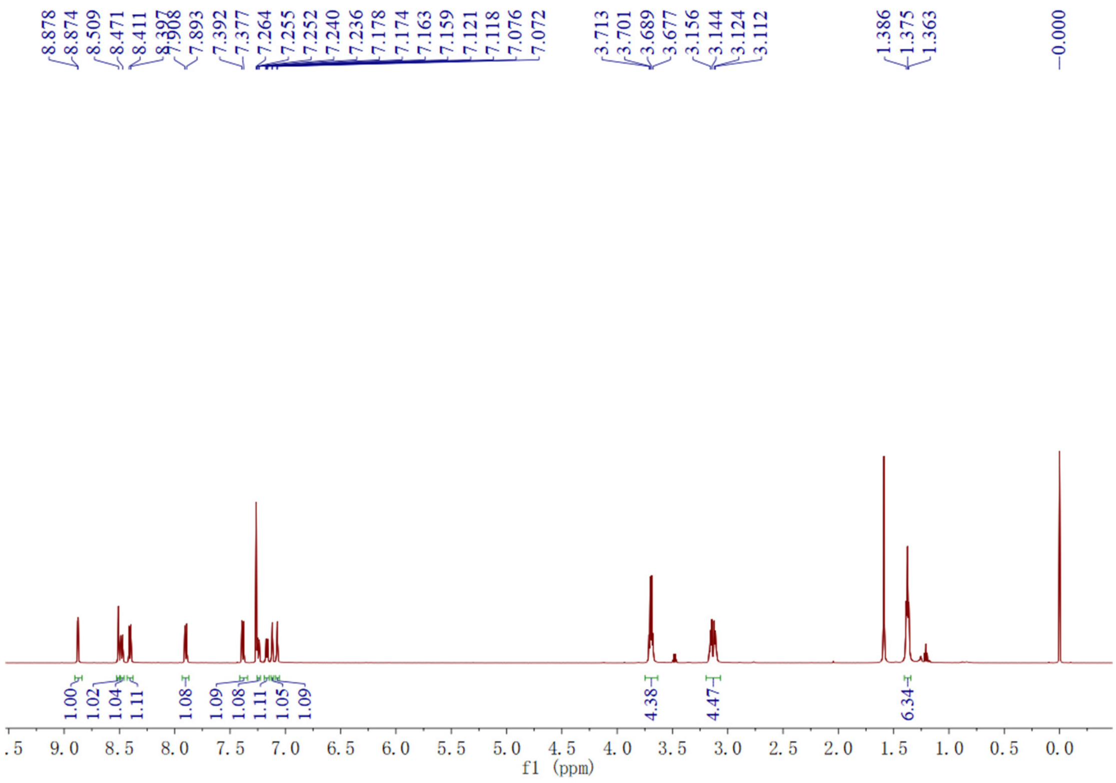

Figure S8. ^{1}H NMR spectrum of **DDX-DNP**.

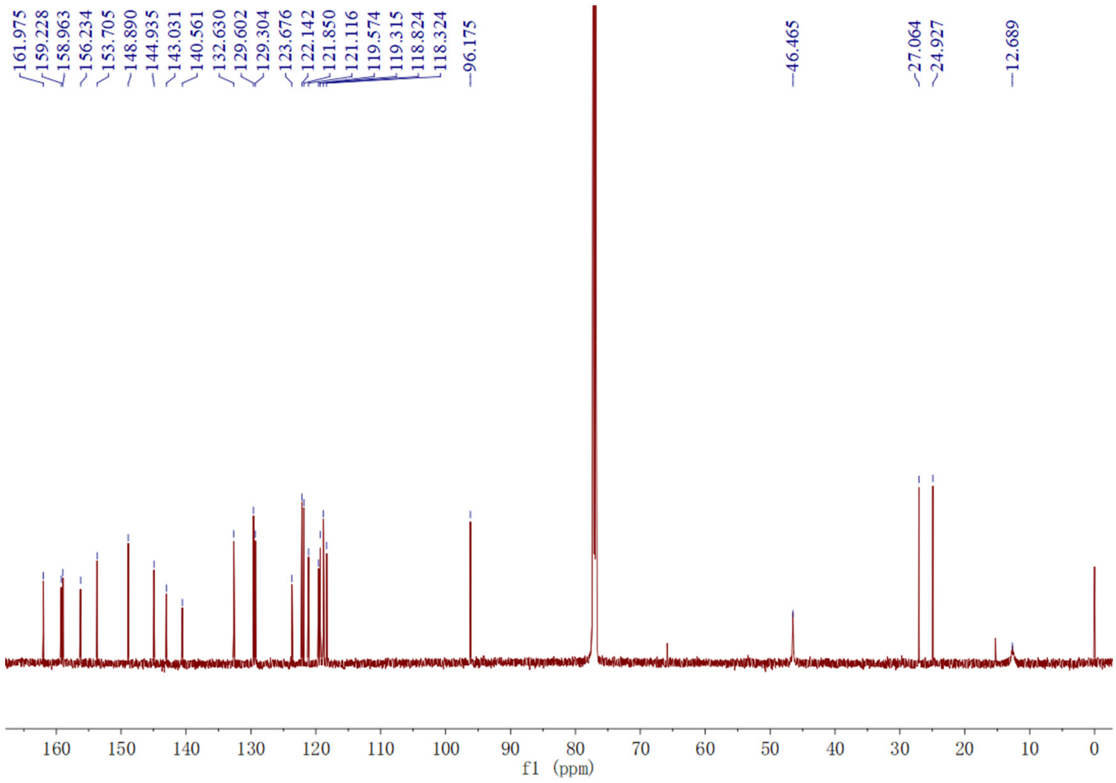

Figure S9. ^{13}C NMR spectrum of **DDX-DNP**.

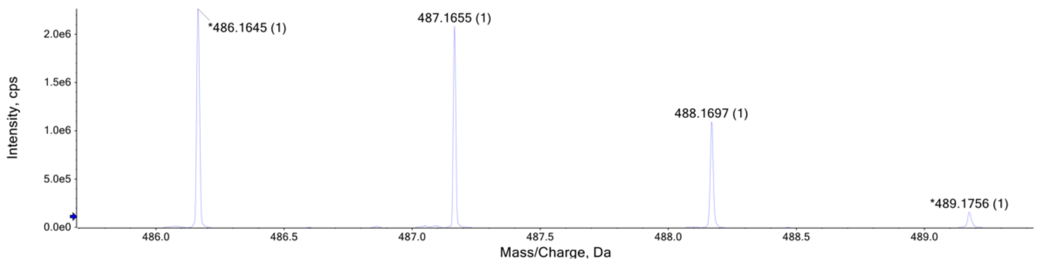

Figure S10. HRMS of **DDX-DNP**.

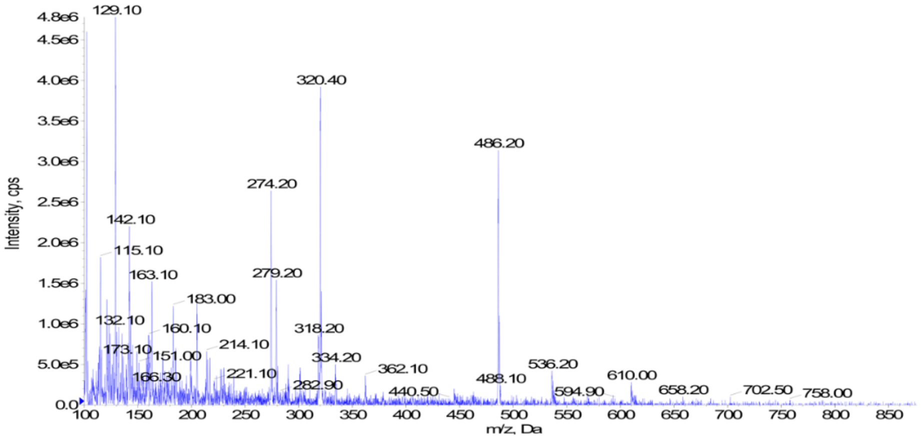

Figure S11. ESI-MS spectra of **DDX-DNP** in the presence of excess amount of NaHS in the phosphate buffer (50 mM, pH 7.4) containing acetonitrile.

Table S1. The tongue coating fungi strains used in this study.

Position	*Strain*	Position	*Strain*
A1	*Sarocladium bactrocephalum*	E5	*[Candida] saitoana*
A2	*Festuca parvigluma*	E6	*Wickerhamiella sorbophila*
A3	*Pichia membranifaciens*	E7	*Rhodotorula sinensis*
A4	*Pichia cecembensis*	E8	*Pichia anomala*
A5	*Candida africana*	E9	*Candida sojae*
A6	*Schizosaccharomyces pombe*	F1	*Citeromyces matritensis*
A7	*Pichia kluyveri*	F2	*Candida sp.*
A8	*Metschnikowia sp.*	F3	*Pichia manshurica*
A9	*Saturnispora hagleri*	F4	*[Candida] fructus*
B1	*Meyerozyma guilliermondii*	F5	*[Candida] boidinii*
B2	*Torulaspora delbrueckii*	F6	*[Candida] glabrata*
B3	*Hanseniaspora uvarum*	F7	*Candida orthopsilosis*
B4	*Pyrus sinkiangensis*	F8	*Candida quercitrusa*
B5	*Guignardia mangiferae*	F9	*Zymoseptoria tritici*
B6	*Wickerhamomyces anomalus*	G1	*Candida albicans*
B7	*Sclerotinia sclerotiorum*	G2	*Debaryomyces hansenii*
B8	*Moellerodiscus lentus*	G3	*Clavispora lusitaniae*
B9	*Pichia fermentans*	G4	*Vicia sativa subsp.*
C1	*Saccharomyces cerevisiae*	G5	*Lodderomyces elongisporus*
C2	*Candida parapsilosis*	G6	*Hyphopichia sp.*
C3	*Fungal sp.*	G7	*Candida tropicalis*
C4	*Kazachstania servazzii*	G8	*Candida metapsilosis*
C5	*Pichia occidentalis*	G9	*Candida xestobii*
C6	*Pichia sp.*	H1	*Meyerozyma caribbica*
C7	*Candida labiduridarum*	H2	*Pichia kudriavzevii*
C8	*Wickerhamiella brachini*	H3	*[Candida] intermedia*
C9	*Candida dubliniensis*	H4	*Jaminaea sp.*
D1	*Wickerhamiella spandovensis*	H5	*[Candida] norvegica*
D2	*Clavispora reshetovae*	H6	*Phaeoacremonium australiense*
D3	*Cystobasidium minutum*	H7	*Cystobasidium lysinophilum*
D4	*Papiliotrema flavescens*	H8	*Dioszegia sp*
D5	*Yarrowia lipolytica*	H9	*Bryochiton sp.*
D6	*Lachancea fermentati*	I1	*Cystobasidium slooffiae*
D7	*Kodamaea ohmeri*	I2	*Erythrobasidium hasegawianum*
D8	*Pucciniomycotina sp.*	I3	*Symmetrospora marina*
D9	*Rhodotorula mucilaginosa*	I4	*Vishniacozyma sp.*
E1	*Cystobasidiomycetes sp.*	I5	*Nolanea sp.*
E2	*Wickerhamiella pararugosa*	I6	*Trichosporon jirovecii*
E3	*Sphaerulina polyspora*	I8	*Aureobasidium pullulans*
E4	*Candida zeylanoides*	I7, I9	*MtB*
